# Integrated Structural, Physiological, and Molecular Assessment of Sugar Beet Infested by *Scrobipalpa ocellatella* Under Field Conditions

**DOI:** 10.3390/antiox15050624

**Published:** 2026-05-14

**Authors:** Ildikó Jócsák, Ádám Csóka, Tamás Donkó, György Végvári, Bálint Gerbovits, Ferenc Csima, Sándor Keszthelyi

**Affiliations:** 1 Institute of Agronomy, Hungarian University of Agriculture and Life Sciences, 7400 Kaposvár, Hungary; gerbovits.balint@phd.uni-mate.hu (B.G.); csima.ferenc.1@phd.uni-mate.hu (F.C.); keszthelyi.sandor@uni-mate.hu (S.K.); 2 Medicopus Nonprofit Ltd., 40 S. Guba Str., 7400 Kaposvár, Hungary; adam.csoka@sic.medicopus.hu (Á.C.); donko.tamas@sic.medicopus.hu (T.D.); 3 Institute of Viticulture and Oenology, Faculty of Natural Sciences, Eszterházy Károly Catholic University, Eszterházy tér 1, 3300 Eger, Hungary; vegvari.gyorgy@uni-eszterhazy.hu

**Keywords:** beet moth, gene expression, insect damage, non-destructive, analysis, physiological response, stress phenomenon

## Abstract

Background: The beet moth, *Scrobipalpa ocellatella* Boyd, 1858 (Lep.: Gelechiidae), is an increasingly important pest whose climate-driven expansion threatens sugar beet (*Beta vulgaris* L.) production in Europe. This study aimed to characterize the structural, physiological, biochemical, and molecular responses of sugar beet to infestation. Methods: Plants were analysed using computed tomography (CT), SPAD and NDVI measurements, HPLC-based sugar analysis, FRAP and MDA assays, and RT-qPCR of antioxidant-related genes. Results: CT imaging enabled non-destructive detection of larvae (mean length: 7.32 ± 0.73 mm) and pest-induced cavities (982.20 ± 316.04 mm^3^). SPAD did not differ significantly among treatments, whereas NDVI was consistently reduced in infested plants, declining from 0.648 ± 0.031 in non-infested plants to 0.593 ± 0.038 in infested-treated plants and 0.611 ± 0.021 in infested-untreated plants at the first sampling. Infestation induced pronounced oxidative stress, with FRAP increasing from 14.102 ± 0.943 to 25.471 ± 0.922 µg AA eq g^−1^ FW and MDA from 558.065 ± 21.819 to 1325.806 ± 16.762 nmol g^−1^ FW in untreated infested plants. Antioxidant gene expression was significantly upregulated, particularly for SOD, CAT, APX, DHAR, MDAR, and GPX. Conclusions: *S. ocellatella* infestation triggered coordinated oxidative stress responses in sugar beet, while CT and NDVI proved useful for early damage detection.

## 1. Introduction

The beet moth, *Scrobipalpa ocellatella* Boyd, 1858 (Lep.: Gelechiidae) is a Mediterranean-fauna element, which is one of the most dangerous pests of sugar beet, *Beta vulgaris* L. in Hungary [1]. In recent years *S. ocellatella* has caused increasing economic damage in Europe and other regions [2,3,4,5], and it is currently one of the most serious threats to sugar beet [4,6,7]. Severe infestations can cause 40–60% yield loss and up to a 2% reduction in sugar content [5,8]; therefore, the significance of diagnostic and control methods [1] have become increasingly important, since infested beets are unsuitable for storage and feeding. The appearance of 75% root rot of S. ocellatella in the Carpathian Basin under open field conditions certainly confirms the northward advance of the species, which can be compared with the change in the abiotic background provided by the warming climate. Previously, the species was registered as a rare, possible pest in Hungarian conditions [9].

As an oligophagous insect [10], its larvae damage cultivated beet species, including sugar beet, fodder beet (*Beta vulgaris* subsp. vulgaris convar. crassa), beetroot (*Beta vulgaris* subsp. vulgaris convar. vulgaris), chard (*Beta vulgaris* subsp. vulgaris convar. cicla) as well as wild beet relatives [8], attacking all vegetative organs and, from May to November, also generative tissues. Low infestation levels cause leaf curling and deformation, whereas during warm, dry periods up to 25–30 larvae per plant may occur, resulting in cigar-shaped, desiccated leaf rosettes and severely deformed canopies [8]. Furthermore, the mastication of the photosynthetic organs, which also appears as the site of damage, confirms the local adaptation of the species, since instead of this form of damage characteristic of Mediterranean areas, masticated damage to the head and shoulders of the sugar beet body dominated in Hungary [11]. The species overwinters mainly as pupae in soil, beet residues, or storage prisms, while advanced larvae may survive in living plant tissues. Adult emergence typically begins in late April or May, with two distinct flight peaks influenced by overwintering strategy [8].

Control relies primarily on agrotechnical measures, including autumn ploughing of beet residues, loss-free harvesting, and adequate irrigation [8]. Pesticide treatment of the prism can also be a technological element against larvae that overwinter and pupate in the soil of the beet prism. Nonetheless, climate change has markedly raised infection pressure during the growing season due to its expanded proliferation [12], consequently, the necessity for protection has intensified. This requires the prompt identification of damage and the investigation of the impacts of both the pest and the active substances employed against it on plants.

From the perspective of sugar beet, it is crucial to investigate methods for promptly detecting *S. ocellatella* damage. Numerous stress recognition and monitoring techniques exist in modern sensor-based agricultural monitoring systems [13]; however, their use for biotic stressors has been limited. This group encompasses non-invasive techniques, including SPAD and NDVI devices, for the swift assessment of photosynthetic status, which have gained popularity in the evaluation of abiotic plant stress [13,14,15,16,17], however are less frequently employed in the context of biotic pests.

Computed-tomography (CT) techniques are largely employed for the accurate monitoring of structural changes in human diagnostics [18,19,20], while they are also utilized in plant imaging [21], albeit at a lesser extent and in a pioneering capacity. The computed-tomography, a non-invasive imaging technique, enables three-dimensional visualisation of the texture and volume of subjects being studied. Despite the extensive utilisation of this technology in the fields of human and animal sciences, it remains less prevalent in plant sciences [22]. This laboratory approach provides objective measurements, thereby facilitating the examination of target organisms in their natural environments without causing disruption to the living organisms [23,24]. In the domain of plant sciences, particularly in the field of agro-entomology, the utilisation of these technologies facilitates the observation of concealed pests in natural condition without influencing their feeding, movement, and development [21,25]. One element of our comprehensive scientific investigation entailed crucial biological data concerning the life cycle of *S. ocellatella*, a relatively lesser researched gelechiid pest of sugar beets. We aimed to fulfill this expectation, among others, by utilizing a computed tomography approach, which would enhance our understanding of this species.

Numerous processes take place in the plasma membrane of plant cells after an attack by herbivores. The reaction begins with the detection of molecular stimuli and effector surfaces, which leads to an increase in cytosolic calcium ([Ca^2+^]cyt) levels, depolarization of the plasma membrane potential (Vm) [26], and activation of mitogen-activated protein kinases (MAPK) [27], nicotinamide adenine dinucleotide phosphate (NADPH) oxidase activation, and the production of reactive oxygen species (ROS) and reactive nitrogen species (RNS) [28]. There are several aspects of how biotic stress caused by insect herbivory affect the host organisms [29], numerous changes in plant primary metabolism occur, such as elevated or decreased photosynthetic efficiency, alterations in anabolic metabolism via relocation of carbon and nitrogen sources triggering complex responses including the formation of reactive oxygen species (ROS), consequently altering plant growth rate and yield as well. ROS accumulation can be detected in plants within 24 h after infection by herbivorous insects, and it was observed that plant ROS and antioxidant production may also be some kind of defense mechanism against pests [30]. Through this, plants can cause oxidative damage to the digestive system of insects and/or participate in the oxidative modification of essential elements of insect nutrition, as has already been demonstrated in *Helicoverpa zea* [31,32], *Spodoptera littoralis* [33] and several aphid species [34]. Although the defensive value of induced secondary metabolites such as nicotine, terpenes, benzoxazinoids, and glucosinolates is well established, the alterations of primary metabolic processes remain insufficiently understood in plants in general [31], and particularly in the case of *S. ocellatella* infestation in sugar beet, where integrative studies linking physiological, structural, and molecular responses are still lacking.

The objectives of this study were to investigate the infestation dynamics and damage progression of *S. ocellatella* in sugar beet under field conditions, and to develop and evaluate non-invasive monitoring approaches for the early detection of biotic stress. For this purpose, structural damage was assessed by computed tomography (CT), including the visualization of larvae, the measurement of larval body length, cavity volume, and Hounsfield Unit values of damaged and intact tissues. Plant physiological status was evaluated by non-invasive SPAD and NDVI measurements, as well as by the determination of leaf dry weight. In addition, the effects of infestation and pesticide treatment on primary metabolism were quantified by HPLC-based analysis of taproot sucrose, glucose, and fructose concentrations. Oxidative stress responses were assessed through the determination of total antioxidant capacity (FRAP) and lipid peroxidation (MDA). Furthermore, the expression patterns of key antioxidant-related genes, including *SOD*, *CAT*, *APX*, *GPX*, *DHAR*, *MDAR*, and *GR*, were analysed to reveal molecular-level responses to infestation. Finally, structural, physiological, biochemical, and molecular datasets were integrated using correlation analysis and principal component analysis (PCA) to improve the understanding of host–pest interactions and to support the development of early diagnostic strategies.

This aims to facilitate a more accurate assessment of the spreading dynamics of this sugar beet pest and the potential level of damage during the initial symptom onset period.

## 2. Materials and Methods

**Figure 1:**
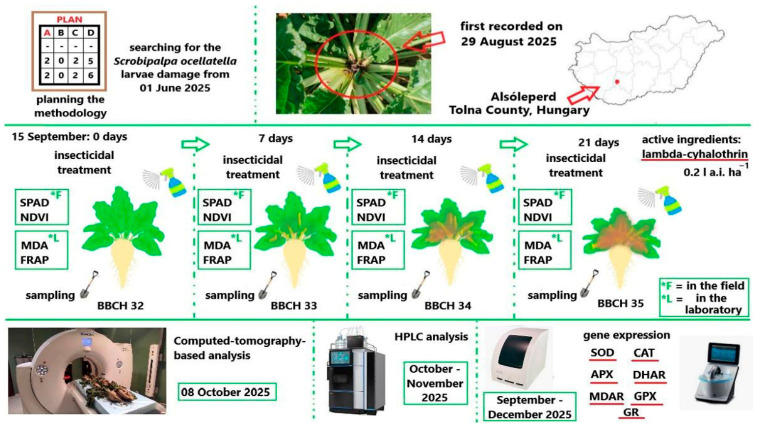
Experimental workflow integrating field-based monitoring, biochemical assays, and molecular analyses to characterize sugar beet responses to beet moth infestation Alsóleperd is the village of observation.

The timeline of the treatments, the sampling, the field and laboratory investigations and outline of the methodology and research structure is presented in Figure 1.

### 2.1. Description of Experimental Field

To objectively understand the damage caused by *S. ocellatella* in sugar beet and the stress phenomena developed on this host plant, we set up a laboratory experiment based on open field surveys in a 52.80 ha (GPS: 46°46′32.14″ N, 18°15′88.78″ E) sugar beet field near Alsóleperd (Hungary, Tolna county). To express the damage caused by *S. ocellatella* in a given year, we first searched for areas in southern Transdanubia where intensive sugar beet cultivation has been taking place for several years and the pest has already appeared with previously detected stable damage. The sugar beet field selected for the study, which we designated as the experimental sampling area, met these prerequisites. The sugar beet hybrid sown in the area was KWS Grizella (KWS SAAT SE & Co. KGaA and KWS LOCHOW GmbH, Einbeck, Germany), which was sown on April 10–12, 2025, at 1.08–1.10 U ha^−1^. The sugar beet crop received nutrient supplementation, insecticidal herbicides, and fungicide treatments to support healthy development at a phenologically justified time, which were implemented until the middle of the tuberization stage of the sugar beet (BBCH 31-35). After that, no chemical treatment or other technological elements aimed at pest control were applied to the crop.

### 2.2. Field Experimental Design and Sampling

**Figure 2:**
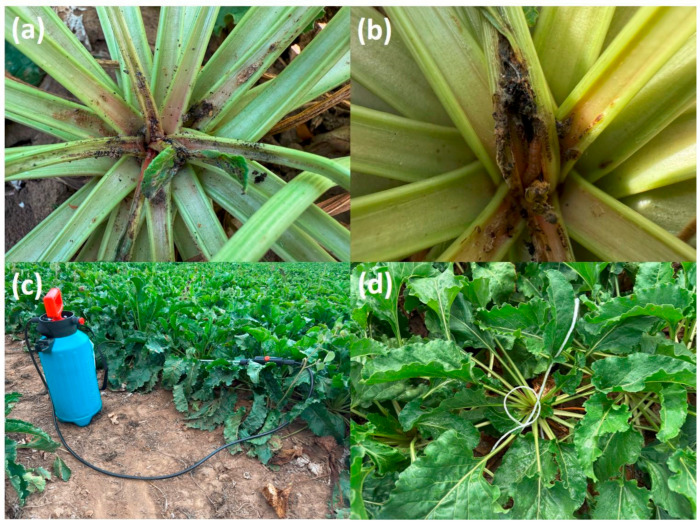
Experimental design and sugar beet sampling before harvesting and laboratory analysis. Damaged sugarbeet plants (**a**). The investigation revealed the presence of vagile larvae (**b**). Treatment with a lambda-cyhalothrin (**c**). The treated plants were marked (**d**).

To objectively determine the damage caused by *S. ocellatella*, we started our laboratory survey based on field sampling on August 29, 2025, at the beginning of the maturation stage (BBCH 40-42). During this time, we noticed the holes made by the pest, which started on the petiole, at the base of the rosette, and in the parts of the sugar beet from the top to the shoulder, due to the masticated damage from the larvae. We identified the damaged plants through visual inspection. We classified as damaged those plants on which the larva could be easily identified by folding the rosette leaves aside or where the mastication accompanying the damage could be observed. The percentage of plant damage caused by *S. ocellatella* in sugar beet was determined by plant analysis per linear meter performed in 3 replicates (Figure 2).

To objectively determine the requirements of the damaging larvae in the plant, depending on the individual development stage, we examined the plant population on five different occasions, with a weekly schedule (September 15, 22, 29, October 06, 2025) following the initial registration of the pest presence (August 29). For each survey, 9 damaged and 9 healthy, pest-free plant individuals were randomly selected within the field after leaving 16.5 m from the edge of the field. We marked these plants with a colored tape attached to the base of the leaf rosettes for the purpose of later sampling. In order to learn about the morphological and physiological changes caused by the damage caused to each plant included in the study at a given recording time, the damage process was blocked by contact insecticide treatment on the day before the recordings (active ingredient: lambda-cyhalothrin 100 g L^−1^, dose 0.2 L ha^−1^, water volume 300 L ha^−1^). The main purpose of the insecticide treatment was to prevent further damage to the plant caused by *S. ocellatella*. The insecticide treatment was applied solely to prevent further feeding activity and to stabilize the level of herbivore damage prior to measurements, and was not considered an experimental treatment factor. The different treatments were represented by samplings that followed each other at the same time interval, representing the progress of the damage process (Figure 1). Thus, during the five recordings, which were inserted at linear intervals, we recorded the SPAD (Soil Plant Analysis Development—SPAD-502; Konica Minolta Sensing Inc., Tokyo, Japan) and NDVI (PolyPen RP 410 instrument (Photon Systems Instruments, Drásov, Czech Republic) values of the marked plants, which indicated the progressive progress of the ongoing damage event. After each open field recording, the plant individuals included in the study were dug up and transported to the plant physiology laboratory of the Department of Agronomy of the Kaposvár Campus of the Hungarian University of Agriculture and Life Sciences.

On each sampling date, samples were collected from three independent biological replicates (*n* = 3 plants per treatment group). For infested plants (both treated and untreated), leaves associated with the masticated petiole were specifically selected to ensure that physiological and biochemical analyses directly reflected the effects of herbivore damage. In the case of healthy control plants, we sampled symptom-free leaves at the same stage of development. We used different amounts and types of plant tissue for each test. We took 1 g of leaf tissue for fresh/dry weight measurement, 0.1 g each for FRAP and MDA assays, 0.03 g for gene expression studies, and 1 g of taproot samples for HPLC studies. After sampling, the plant materials were stored at −20 °C, while the samples for gene expression studies were stored at −80 °C.

### 2.3. The Methodology of Computer Tomography Imaging

**Figure 3:**
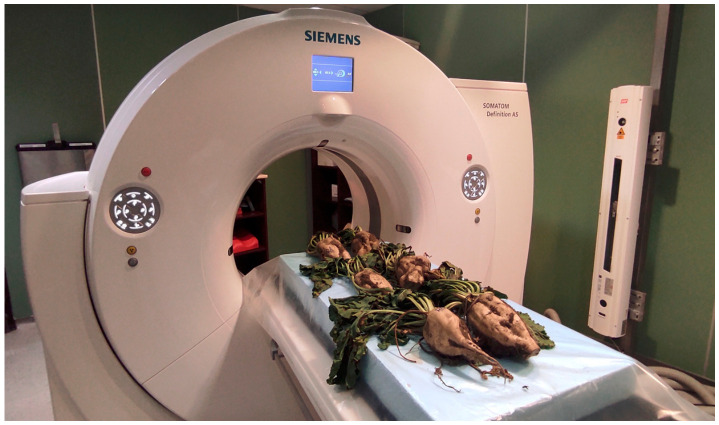
The analysed sugar beet samples in the Siemens CT scanner. The acquisition parameters with a 100 kV tube voltage, with an X-ray dose of 250 mAs, spiral data collection mode with a pitch of 0.4, slice thickness of 0.6 mm, field of view (FoV) of 102 mm, and convolution kernel were I50f. The data collection takes 60–90 s, so the scan did not exceed 10 min for the sugar beet samples analysed. The total number of reconstructed CT images ranges from approximately 1000 to 7000, depending on sample size and resolution.

In the laboratory, we analyzed 10-10 plants that we collected from the field in the experimental sampling. The computer tomography analysis and parameter methods used in the imaging were determined based on several of our previous studies [21,35]. The collected sugar beet samples were placed on the examination table, which was fixed in the polystyrene cell. The plants were then scanned using a Siemens SOMATOM Definition AS+ CT scanner (Siemens, Erlangen, Germany) (Figure 3).

**Figure 4:**
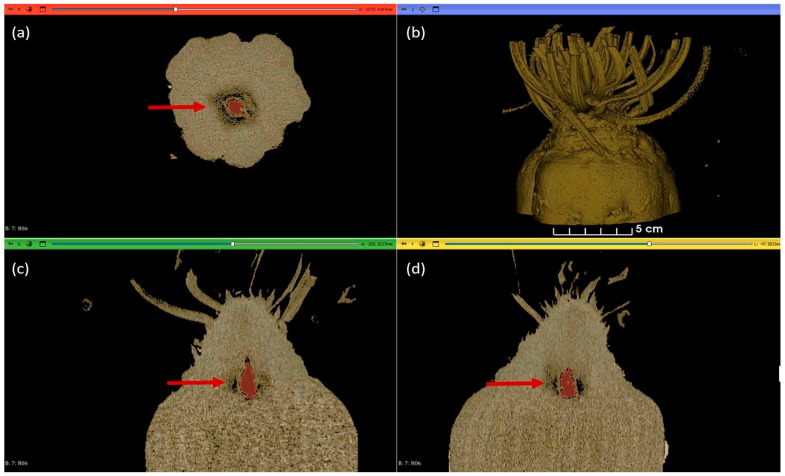
The Computed−tomography based visualisation of the collected and analysed sugar beet samples with Slicer 5.8.1. software. Axial (**a**). 3D (**b**). Coronal (**c**). Sagittal (**d**). The formed cavity is indicated by a red arrow. The parameters include the number of larvae per plant, the longitudinal size of the larvae (mm), the volume of the formed cavity (mm^3^), and the Hounsfield Unit of the damaged plant parts and the healthy ones.

The analyzed sugar beet CT images were segmented using Slicer 5.8.1 software (Slicer Community, Harvard Medical School, Boston, MA, USA) [36]. During the analysis, the Segment Editor module of this software was used to segment the parameters to be analyzed using the Erase, Draw, Paint, and Threshold tools. The 3D visualization was performed using the CT-AAA, CT-AAA2, and CT-MIP presets of the Volume Rendering module. The information from the selection is collected and presented in tabular and graphical form by the Segment Statistics module (Figure 4).

### 2.4. Non-Invasive Physiological Measurements

#### 2.4.1. Chlorophyll Content Estimation by Means of SPAD Index Measurement

The relative chlorophyll content of plants was measured with a Minolta SPAD 502 (Konica Minolta, Europaallee, 17 30855 Langenhagen, Germany). It can measure the intact transmission of plants at wavelengths between 650 and 940 nm. The chlorophyll content and the SPAD index value are closely correlated, and therefore the result of the measurement provides an estimate of the chlorophyll content. Measurements are made by briefly enclosing the leaf inside the sensor, which is 2 × 3 mm. Three plants per treatment were measured; 10 values for each plant were collected from different parts of the leaf surface.

#### 2.4.2. Determination of the Normalized Difference Vegetation Index (NDVI)

The Normalized Difference Vegetation Index (NDVI) was used as a non-invasive indicator of plant physiological status, reflecting the relative intensity of green biomass. NDVI is based on the differential absorption and reflectance properties of plant tissues. whereby chlorophyll pigments in healthy leaves strongly absorb visible red light, while the internal leaf structure reflects a substantial proportion of near-infrared radiation. NDVI measurements were performed using a PolyPen RP 410 instrument (Photon Systems Instruments, Drásov, Czech Republic). The selected leaf was positioned under the leaf clip of the device, which simultaneously measures reflectance in the red and near-infrared spectral regions. Measurements were initiated by a single-button operation, following a procedure analogous to that used for SPAD index determination. The instrument automatically calculated NDVI values based on the recorded reflectance data.

### 2.5. HPLC Analyses

#### 2.5.1. Sample Preparation

A sample of 1 g was taken from fresh sugar beet taproot (chopped). The sample was ground in a mortar with the addition of 10 mL of distilled water. The extract was then placed in a 25 mL centrifuge tube. The sample was vortexed for 30–60 s, then centrifuged at 4 °C at 15,000 rpm in a Hettich Mikro 22R ultracentrifuge (Andreas Hettich GmbH, Föhrenstraße 12, Tuttlingen, Germany), after which the supernatant was passed through a 0.45 µm Millipore Syringe Filter Unit SLHN-13 purchased from Waters Corporation (Milford, MA, USA).

#### 2.5.2. Sugar Content Determination by HPLC

The WATERS (WATERS Co., 34 Maple Street, Milford, MA 01757, USA) HPLC system had the following components: 2487 Dual λ absorbance detector for ascorbic acids, 2414 RI detector for sugar determinations, a 1525 Binary HPLC pump, a column thermostat (30 °C), a 717 plus autosampler (set to 5 °C), and an in-line degasser; the equipment is controlled using EMPOWERTM2 software (v.2154.2). Determination of sugar components Standards of glucose [CAS 50-99-7], fructose [CAS 57-48-7], sucrose [CAS 57-50-1], sorbitol [CAS 50-70-4], and mannitol [CAS 69-65-8] were obtained from Sigma-Aldrich Chemical Co. (St. Louis, MO, USA). A WATERS 2414 Refractive Index Detector was installed, and the flow cell temperature was set to 40 °C. The chromatographic separation was performed on a WATERS Sugar-PakI column (300 mm × 6.5 mm ID), tempered to 90 °C. The protection of the column was carried out with the special in-line filter for sugar. The sampling frequency was 10/s with a sensitivity of 256. The mobile phase was water, in which 50 mgL^−1^ Ca-EDTA (calcium disodium ethylene diamine tetraacetate) was dissolved and filtered by Millex 0.45 µm vacuum filter. The flow rate was 0.5 cm^3^ min^−1^, resulting in a pressure of 450 ± 20 psi on the column. The injected sample quantity was 20 µL, with a running time of 30 min. Retention times of the individual components were sucrose 8.4 min, glucose 10.2 min, fructose 11.7 min.

### 2.6. Determination of Oxidative Stress Markers

#### 2.6.1. Total Antioxidant Capacity (FRAP Assay)

Total antioxidant capacity was measured using the ferric reducing antioxidant power (FRAP) assay based on the method by Benzie and Strain [37], following the steps for estimating total antioxidant activity. The FRAP working solution was made fresh by mixing three ingredients in a 10:1:1 ratio: acetate buffer (300 mM, pH 3.6), 2,4,6-tripyridyl-s-triazine (TPTZ; 10 mM) and 20 mM FeCl_3_ × 6 H_2_O. The resulting working solution is light-sensitive and was therefore stored in the dark until use. Sample extraction was performed as follows: leaf tissues were homogenized in 1.5 mL of phosphate buffer (pH 7.6), then homogenates were transferred into 2 mL microcentrifuge tubes and centrifuged at 13,000 rpm for 10 min at 4 °C using a refrigerated centrifuge (MIKRO 220R, Andreas Hettich GmbH & Co. KG, Tuttlingen, Germany). For the FRAP assay, 1950 µL of the freshly made working solution was put into 2 mL microcentrifuge tubes, and then 50 µL of the sample supernatant was quickly added to start the reaction evenly. The reaction mixtures were immediately incubated at 37 °C for 15 min. Absorbance was subsequently measured at 593 nm using a spectrophotometer. Total antioxidant capacity was expressed as micrograms of ascorbic acid equivalents per gram of fresh weight (µg AA eq g^−1^ FW). All measurements were performed in triplicate, and results are presented as mean values.

#### 2.6.2. Lipid Peroxidation (MDA Content)

Lipid peroxidation was quantified by determining malondialdehyde (MDA) content using the thiobarbituric acid (TBA) assay according to Heath and Packer [38], with minor modifications. Fresh plant tissue (0.1 g) was homogenized in a pre-cooled mortar using 1.5 mL of 0.1% (*w*/*v*) trichloroacetic acid (TCA). The homogenates were transferred into 2 mL microcentrifuge tubes and centrifuged at 13,000 rpm for 10 min at 4 °C using a refrigerated centrifuge (MIKRO 220R, Andreas Hettich GmbH & Co. KG, Tuttlingen, Germany). The reaction reagent consisted of 20% (*w*/*v*) TCA containing 0.5% (*w*/*v*) thiobarbituric acid (TBA). Following centrifugation, 0.4 mL of the clear supernatant was mixed with 1.6 mL of the TCA–TBA reagent. The reaction mixtures were incubated in screw-capped tubes at 96 °C for 30 min and subsequently cooled to room temperature. Absorbance was measured spectrophotometrically at 532 nm, with non-specific turbidity corrected by subtracting absorbance values measured at 600 nm. MDA concentration was expressed as nanomoles per gram fresh weight (nmol g^−1^ FW). All measurements were conducted in triplicate, and results are presented as mean values.

### 2.7. Gene Expression Analysis

#### 2.7.1. RNA Isolation and cDNA Synthesis

Total RNA was isolated from leaf tissue samples (30 mg fresh weight) using the RNeasy Tissue Mini Kit (Qiagen, Germantown, MD, USA). Samples were homogenized in lysis buffer using a TissueLyser II high-throughput homogenizer (Qiagen, Germantown, MD, USA). To minimize RNA degradation, the homogenizer adapter was pre-cooled to −20 °C prior to sample processing. RNA extraction was carried out strictly according to the manufacturer’s instructions. The concentration and purity of the isolated RNA were assessed using a NanoDrop™ One Microvolume UV–Vis spectrophotometer (Thermo Scientific™, Waltham, MA, USA). Genomic DNA contamination was eliminated by on-column DNase digestion, performed at 42 °C for 2 min. Subsequently, first-strand cDNA synthesis was carried out using the QuantiTect Reverse Transcription Kit (Qiagen, Germantown, MD, USA) at 42 °C for 15 min, following the manufacturer’s protocol.

#### 2.7.2. RT-PCR Conditions and Relative Gene Expression Calculation

**Figure 5:**
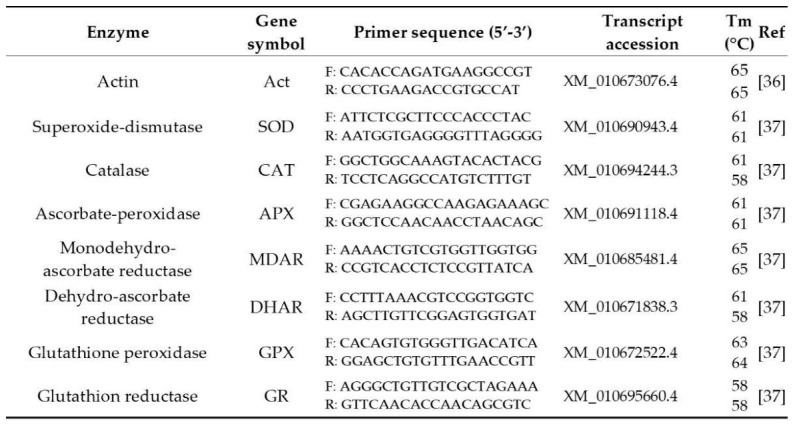
Primers used for gene expression studies.

Quantitative real-time PCR (qPCR) was performed in a Stratagene Mx3000P thermocycler (Agilent Technologies, Santa Clara, CA, USA), using gene-specific primers listed in Figure 5. Gene expression was quantified by real-time PCR using SYBR Green detection chemistry in a quantitative PCR system.

Each reaction contained SYBR Green master mix, gene-specific primers, diluted cDNA template, and nuclease-free water in the final reaction volume. Amplification reactions were conducted under the following thermal cycling conditions: initial denaturation at 95 °C for 10 min, followed by 40 cycles of denaturation at 95 °C for 30 s, annealing at 60 °C for 10 s, and extension at 72 °C for 1 min. Three independent biological replicates were used per treatment and sampling date (n = 3), and each biological replicate was analysed in three technical replicates. Amplicon specificity was verified by melting curve analysis for each primer pair, confirming the amplification of a single, gene-specific product. Primer amplification efficiencies were determined using standard curves generated from serial dilutions of pooled cDNA samples. The amplification efficiency (E) was calculated and only primer pairs with efficiencies between 90–110% and correlation coefficients (R^2^ > 0.99) were used for expression analysis.

Relative gene expression levels were calculated from threshold cycle (Ct) values using the efficiency-corrected 2^−ΔΔCt^ method described by Livak and Schmittgen [39]. This method made it possible to compare the levels of gene expression between treatments and genes of interest in a quantitative way. Relative expression values were subsequently log_10_-transformed to stabilize variance and to improve normality assumptions prior to statistical analyses.

### 2.8. Data Analysis

The experimental unit was an individual plant (biological replicate; n = 3 per treatment and sampling date). For non-invasive measurements (SPAD, NDVI), 15 readings per plant were averaged to avoid pseudo-replication, ensuring that all variables in integrative analyses were based on independent biological units. Each parameter was analyzed separately by sampling date, and temporal effects were evaluated within each treatment using sampling date (Date) as the independent variable. Assumptions of normality (Shapiro–Wilk) and homogeneity of variances (Levene’s test) were assessed prior to analysis (Table S1). When met (*p* ≥ 0.05), one-way ANOVA followed by Tukey HSD was applied; otherwise, Kruskal–Wallis with Dunn’s post hoc test or heteroscedasticity-robust ANOVA (Type III, HC3) with emmeans-based pairwise comparisons was used. p-values were adjusted for multiple comparisons using Tukey or Holm correction as appropriate. Temporal effects were further analyzed using linear models with HC3 covariance estimation. Significant differences were visualized using compact letter displays or bracket annotations with asterisks (* *p* < 0.05, ** *p* < 0.01, *** *p* < 0.001), while non-significant comparisons were omitted. Relationships among variables were assessed using Spearman’s rank correlation and visualized as hierarchically clustered heatmaps. Principal component analysis (PCA) was performed on standardized (z-score) data to explore multivariate response patterns, with interpretation focused on the first two components. Treatment separation in PCA space was statistically evaluated using PERMANOVA based on Bray–Curtis distances (999 permutations), performed separately for each sampling date. As transcriptomic data were not available for all dates, integrative analyses including gene expression were restricted to 15 September, 1 October, and 8 October 2025; accordingly, PCA was conducted on the full dataset, whereas PERMANOVA was applied to this subset. All statistical analyses and visualizations were performed in R (R Core Team; R 4.5.2) and RStudio (R3.6.0+) using the packages readxl, dplyr, stringr, openxlsx, ggplot2, emmeans, pheatmap, and vegan.

## 3. Results

### 3.1. Physiological Responses Assessed by Non-Invasive Methods

#### 3.1.1. Computer-Tomography-Based Imaging

Computer-tomography-based imaging facilitated the non-invasive visualisation of larvae within damaged sugar beet samples, along with their size and the volume of the cavities they had created.

**Figure 6:**
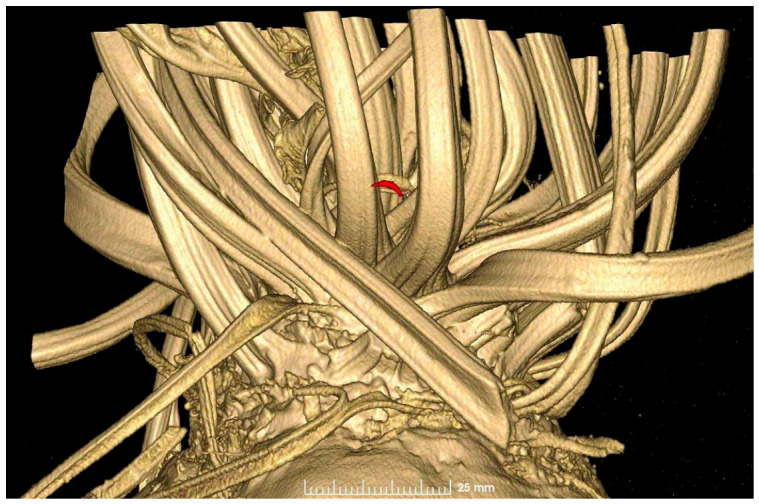
CT-based imaging of sugar beet samples showing *S. ocellatella* larvae (highlighted in red) located in the leaf axis.

*S*. *ocellatella* larvae were identified in multiple instances within the leaf axils of sugar beets, where they were found to be concealed in eight samples of the collected plants (Figure 6). The mean size of *S*. *ocellatella* larvae damaging sugar beet, as determined in laboratory tests, was 7.32 ± 0.73 mm (lower limit: 2.66 mm; upper limit: 9.54 mm).

**Figure 7:**
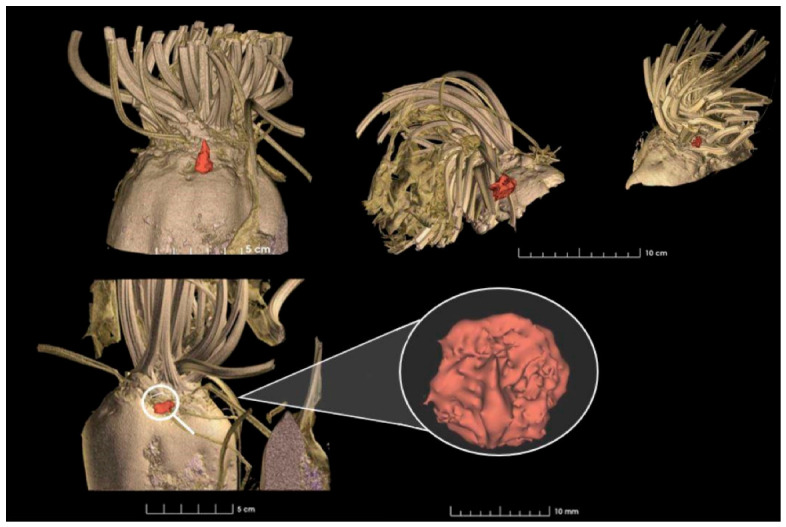
Visualization of cavities formed by *S. ocellatella* using computer-tomography-based Slicer 5.8.1 software.

The mean volume of the cavity formed by *S*. *ocellatella* was 982.20 mm^3^ ± 316.04 (lower limit: 285.464 mm^3^; upper limit: 2855.2 mm^3^) in the analysed samples. The presence of this pest-induced cavity has also been observed in numerous cases within the beet head (Figure 7). The formation of a cavity was observed in all samples as a result of the damage caused by *S*. *ocellatella*, yet the presence of larvae was not detected, a finding that is likely associated with the repellent effect of the pyrethroid derivative lambda-cyhalothrin.

Moreover, the employment of computer tomography imaging facilitated the determination of the Hounsfield Unit values of both damaged and intact plant components. In the case of cavities formed in the beet head and petiole, the average Hounsfield Unit values were −655.93 ± 66.45, while for intact plant parts, the values were 83.94 ± 3.06.

#### 3.1.2. Changes in SPAD and NDVI Values

**Figure 8:**
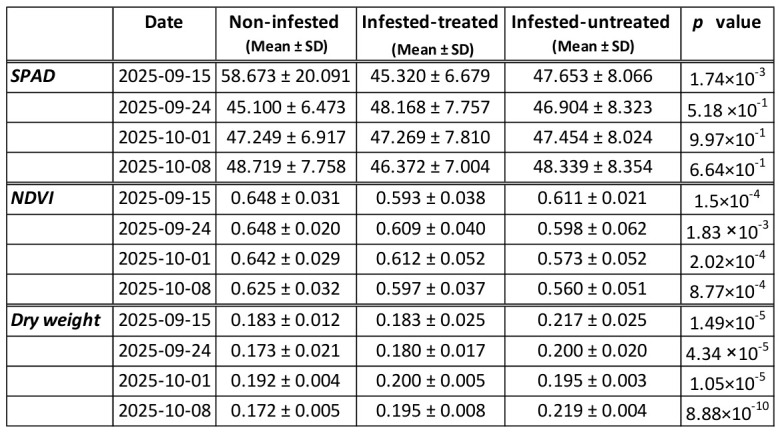
Temporal dynamics of SPAD (n = 15), NDVI (n = 15), and leaf dry weight (n = 3) response to beet moth (*S. ocellatella* Boyd.) infestation and lambda-cyhalothrin insecticide treatment. Values represent mean ± SD (n = 3) measured at four sampling dates during the late growing season (15 September−8 October 2025). Treatments included non-infested control, infested plants treated with insecticide (infested-treated), and infested plants without insecticide protection (infested-untreated).

The chlorophyll content estimation SPAD values did not differ significantly among treatments at any sampling date (robust ANOVA HC3, *p* > 0.05) (Figure 5). However, NDVI differed significantly among treatments at all sampling dates. On 2025-09-15, NDVI was highest in non-infested plants (0.648 ± 0.031), followed by infested-untreated plants (0.611 ± 0.021) and infested-treated plants (0.593 ± 0.038) (*p* = 1.50 × 10^−4^). On 2025-09-24, values were 0.648 ± 0.020 (non-infested), 0.609 ± 0.040 (infested-treated), and 0.598 ± 0.062 (infested-untreated) (*p* = 1.83 × 10^−3^). On 2025-10-01, NDVI values were 0.642 ± 0.029 (non-infested), 0.612 ± 0.052 (infested-treated), and 0.573 ± 0.052 (infested-untreated) (*p* = 2.02 × 10^−4^; infested-treated plants showed intermediate values and were not significantly different from either group). On 2025-10-08, values were 0.625 ± 0.032 (non-infested), 0.597 ± 0.037 (infested-treated), and 0.560 ± 0.051 (infested-untreated) (classical ANOVA, *p* = 8.77 × 10^−4^; infested-treated plants did not differ significantly from non-infested plants) (Figure 8).

### 3.2. Changes in Fresh-Dry Weight

Leaf dry weight (g g^−1^ FW) differed significantly among treatments at multiple sampling dates, but the pattern was most pronounced at the final sampling date. On 2025-10-08, the highest values were recorded in infested-untreated plants (0.219 ± 0.004), followed by infested-treated plants (0.195 ± 0.008), while the lowest values were measured in non-infested plants (0.172 ± 0.005) (Kruskal–Wallis test, *p* = 8.88 × 10^−10^) (Figure 8).

Dry weight varied significantly over time in all treatments. In infested-treated plants, values changed from 0.183 ± 0.005 to 0.197 ± 0.001 (*p* = 2.64 × 10^−7^). Infested-untreated plants also showed a significant temporal effect (0.217 ± 0.005 to 0.220 ± 0.000; *p* = 9.01 × 10^−5^). In non-infested plants, the temporal effect was also significant (*p* = 4.39 × 10^−14^).

### 3.3. Changes in Taproot Sugar Content

**Figure 9:**
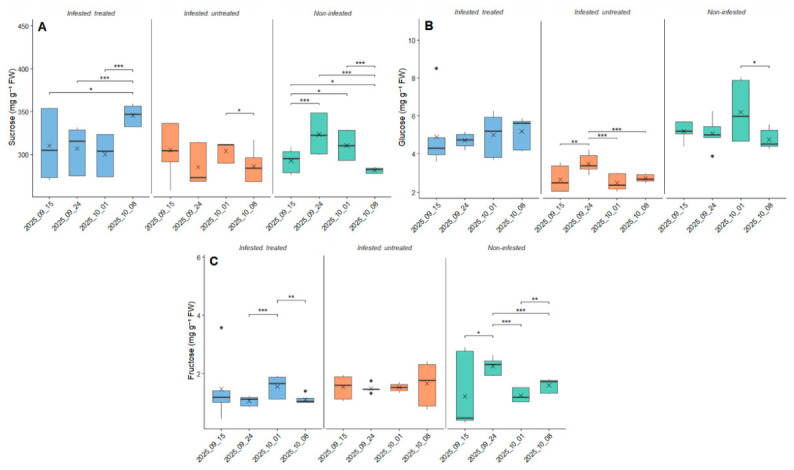
Sucrose (mg g^−1^ FW) (**A**), glucose (mg g^−1^ FW) (**B**), and fructose (mg g^−1^ FW) (**C**) concentrations measured in sugar beet (*Beta vulgaris* L.) taproots under three conditions (infested-treated, infested-untreated, and non-infested) following infestation by the beet moth (*S. ocellatella* Boyd.). The plants were treated with 0.1 L ha^−1^ lambda-cyhalothrin contact active ingredient. Measurements were conducted on four sampling dates (15 September 2025, 24 September 2025, 1 October 2025, and 8 October 2025). Boxplots show median, interquartile range, and data distribution, with × indicating the mean. Statistical differences were evaluated within each treatment across sampling dates using robust ANOVA (HC3) followed by estimated marginal means (emmeans) pairwise comparisons. Significant differences are indicated by horizontal brackets with asterisks (* *p* < 0.05, ** *p* < 0.01, *** *p* < 0.001), while non-significant comparisons are not shown.

The sucrose concentration in sugar beet taproots showed temporal variation within treatments (Figure 9A). At the first sampling, sucrose concentrations ranged from 292.8 mg g^−1^ FW in non-infested plants to 310.3 mg g^−1^ FW in infested-treated plants. Within the infested-treated group, sucrose levels significantly increased by the final sampling date (2025_10_08) compared to all earlier time points (*p* = 0.016–1.45 × 10^−6^). In the infested-untreated group, a significant difference was detected only between 2025_10_01 and 2025_10_08 (*p* = 0.031)**.** In contrast, the non-infested plants showed significant temporal changes, including a decrease between 2025_09_24 and 2025_10_08 (*p* = 2.00 × 10^−7^) and between 2025_10_01 and 2025_10_08 (*p* = 9.93 × 10^−7^).

Glucose concentration showed pronounced temporal variation within treatments rather than consistent differences among treatments at individual sampling dates (Figure 9B). In non-infested plants, the average glucose content decreased from 5.207 mg g^−1^ FW (2025_09_15) to 4.747 mg g^−1^ FW (2025_10_08). A significant difference was detected between 2025_10_01 and 2025_10_08 (*p* = 0.0133). In the infested-treated population, glucose concentration increased slightly over time (from 4.913 to 5.188 mg g^−1^ FW); however, no significant temporal differences were detected within this treatment (*p* > 0.05). In the infested-untreated group, glucose values remained within a lower range (2.647–3.487 mg g^−1^ FW). Significant temporal differences were observed between 2025_09_15 and 2025_09_24 (*p* = 0.00465), as well as between 2025_09_24 and 2025_10_01 (*p* = 8.97 × 10^−6^) and 2025_09_24 and 2025_10_08 (*p* = 3.19 × 10^−5^).

Fructose concentration also exhibited time-dependent patterns within treatments (Figure 9C). In the infested-treated plants, fructose levels decreased between 2025_09_24 and 2025_10_01 (*p* = 4.50 × 10^−4^), followed by a significant increase between 2025_10_01 and 2025_10_08 (*p* = 0.0013). In non-infested plants, fructose concentrations increased significantly from 2025_09_15 to 2025_09_24 (*p* = 0.037), followed by a strong decline between 2025_09_24 and 2025_10_01 (*p* = 1.87 × 10^−11^) and between 2025_09_24 and 2025_10_08 (*p* = 1.83 × 10^−6^). Additionally, a significant decrease was observed between 2025_10_01 and 2025_10_08 (*p* = 0.00187). No significant temporal changes were detected within the infested-untreated group.

### 3.4. Oxidative Status of Sugar Beet

#### 3.4.1. FRAP Responses Across Treatments and Time Points

FRAP values displayed a highly significant treatment effect at all sampling dates, indicating a pronounced alteration in antioxidant capacity. At the first sampling date (2025-09-15), the treatment effect was highly significant (*p* = 2.75 × 10^−34^). FRAP values were 14.102 ± 0.943 µg AA eq g^−1^ FW in non-infested plants, compared with 19.699 ± 1.231 in infested-treated plants and 25.471 ± 0.922 in infested-untreated plants. Infestation was thus associated with a strong elevation in total antioxidant capacity. This pattern persisted on 2025-09-24 (*p* = 2.52 × 10^−21^), with means of 13.945 ± 0.643 (non-infested), 16.949 ± 1.254 (infested-treated), and 20.954 ± 1.755 (infested-untreated).

**Figure 10:**
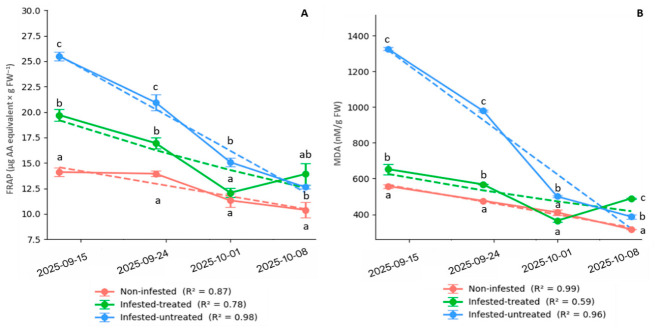
Ferric reducing ability of plasma (FRAP; µg AA equivalent × g^−1^ FW) (**A**) and malondialdehyde content (MDA; nM × g^−1^ FW) (**B**) measured in sugar beet (*Beta vulgaris* L.) leaves under three conditions (infested-treated, infested-untreated, and non-infested) following infestation by the beet moth (*S. ocellatella* Boyd.). The plants were treated with 0.1 L ha^−1^ lambda-cyhalothrin contact active ingredient. Measurements were conducted on four sampling dates (19 September 2025, 24 September 2025, 1 October 2025, and 8 October 2025). Bars represent mean ± SE. Different lowercase letters indicate significant differences among sampling dates within each treatment (*p* < 0.05).

By 2025-10-01, treatment differences remained significant (*p* = 5.27 × 10^−14^). Values decreased overall but retained the same ranking: 11.305 ± 1.486 (non-infested), 12.076 ± 1.060 (infested-treated), and 15.072 ± 0.990 (infested-untreated). At the final sampling date (2025-10-08), the treatment effect persisted (*p* = 2.36 × 10^−7^). Means were 10.138 ± 1.081 (non-infested), 14.008 ± 2.253 (infested-treated), and 12.631 ± 0.408 (infested-untreated). Across all dates, infestation caused elevated FRAP levels, especially in infested-untreated plants, suggesting activation of antioxidant defenses in response to oxidative stress (Figure 10A). FRAP values showed a decreasing trend over time in all treatments.

The regression fit was strongest in infested-untreated plants (R^2^ = 0.98), followed by non-infested plants (R^2^ = 0.87), while infested-treated plants showed a lower fit (R^2^ = 0.78). FRAP showed a strong temporal decrease in all treatments. In infested-treated plants, FRAP declined from 19.702 ± 0.255 to 13.901 ± 0.504 (*p* = 3.03 × 10^−28^). In infested-untreated plants, the decrease was more pronounced, from 25.472 ± 0.190 to 12.630 ± 0.085 (*p* = 2.01 × 10^−51^). In non-infested plants, FRAP decreased from 14.100 ± 0.195 to 10.386 ± 0.342 (*p* = 1.90 × 10^−15^).

#### 3.4.2. MDA Accumulation as a Marker of Lipid Peroxidation

MDA content exhibited highly significant treatment effects at every sampling date, reflecting strong lipid peroxidation under infestation. At 2025-09-15, the treatment effect was highly significant (*p* = 6.10 × 10^−55^). MDA values were 558.065 ± 21.819 nmol g^−1^ FW in non-infested plants, compared with 664.516 ± 16.993 in infested-treated plants and 1325.806 ± 16.762 in infested-untreated plants. The second sampling date (2025-09-24) showed an even stronger separation among treatments (*p* = 2.94 × 10^−65^), with values of 475.807 ± 2.793 (non-infested), 567.742 ± 2.794 (infested-treated), and 979.032 ± 10.073 (infested-untreated). By 2025-10-01, significant differences persisted (*p* = 6.38 × 10^−29^). Means were 411.290 ± 31.730 (non-infested), 364.516 ± 18.319 (infested-treated), and 500.000 ± 10.073 (infested-untreated). At the final assessment (2025-10-08), treatment differences remained highly significant (*p* = 3.87 × 10^−48^). MDA values were 316.129 ± 2.794 (non-infested), 488.709 ± 8.381 (infested-treated), and 387.097 ± 25.604 (infested-untreated). Taken together, infestation caused a pronounced increase in lipid peroxidation across the experimental period, particularly in infested-untreated plants. MDA values also decreased across the sampling period. The regression fit was highest in non-infested plants (R^2^ = 0.99), followed by infested-untreated plants (R^2^ = 0.96), whereas infested-treated plants showed a lower fit (R^2^ = 0.59) (Figure 10B).

MDA showed a strong temporal decrease in all treatments. In infested-treated plants, MDA decreased from 652.52 ± 13.62 to 488.71 ± 1.83, with the lowest value on 2025_10_01 (364.52 ± 4.00; *p* = 1.80 × 10^−51^). In infested-untreated plants, MDA declined markedly from 1325.81 ± 3.66 to 387.10 ± 5.59 (*p* = 4.51 × 10^−82^). In non-infested plants, MDA decreased from 558.07 ± 4.76 to 316.13 ± 0.61 (*p* = 1.46 × 10^−77^).

### 3.5. Antioxidant Enzyme Gene Expression

The selected genes represent key components of the plant antioxidant network and collectively cover its major cellular compartments and functional steps. SOD catalyzes the dismutation of superoxide radicals in chloroplasts, mitochondria and the cytosol, while CAT mainly removes H_2_O_2_ in peroxisomes. APX participates in H_2_O_2_ detoxification primarily in chloroplasts and the cytosol. In addition, GR, DHAR and MDAR function in chloroplasts, mitochondria and the cytosol as enzymes of the ascorbate–glutathione cycle, ensuring the regeneration of antioxidant metabolites.

#### 3.5.1. Expression of Antioxidative Enzymes

**Figure 11:**
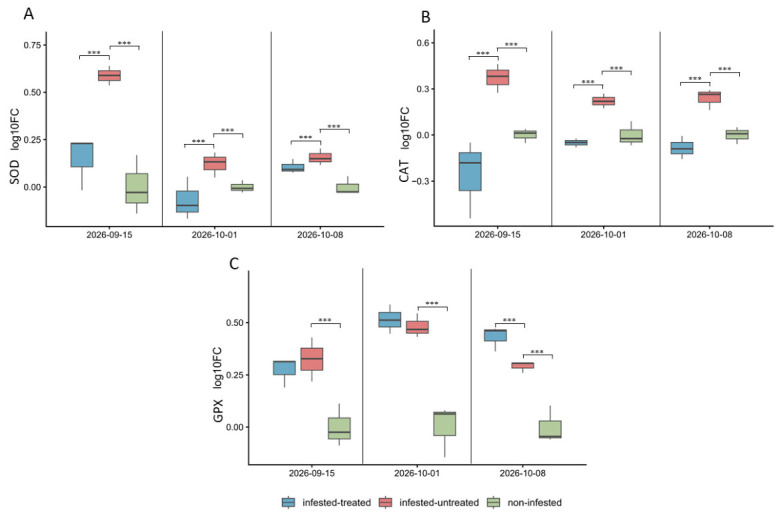
Temporal dynamics of antioxidant gene expression in sugar beet leaves in response to insect infestation and treatment. Relative transcript levels of SOD (**A**), CAT (**B**) and GPX (**C**) are shown as log_10_ fold-change (log_10_FC) values across three sampling dates (15 September, 1 October, and 8 October 2025). Three plant conditions were compared: infested–treated, infested–untreated, and non-infested plants. Gene expression levels were calculated using the efficiency-corrected 2^−ΔΔCt^ method and normalized to the reference gene actin (ACT). Boxplots represent biological replicates (n = 3), with the median indicated by the horizontal line within each box, boxes representing the interquartile range, and whiskers indicating the full data range. Each biological replicate consisted of pooled leaf samples collected from individual plants per treatment and sampling date. Statistical differences were evaluated within each treatment across sampling dates using robust ANOVA (HC3) followed by estimated marginal means (emmeans) pairwise comparisons. Significant differences are indicated by horizontal brackets with asterisks (* *p* < 0.05, ** *p* < 0.01, *** *p* < 0.001), while non-significant comparisons are not shown.

At the first sampling date, SOD expression was significantly higher in infested-untreated plants (0.590 ± 0.050) than in both infested-treated (0.147 ± 0.144) and non-infested plants (1.16 × 10^−16^ ± 0.157) (*p* = 9.38 × 10^−22^), while the latter two treatments did not differ significantly from each other (Figure 11A). For CAT, infested-untreated plants (0.370 ± 0.095) also showed significantly higher expression compared to that of infested-treated plants (−0.257 ± 0.254) and non-infested plants (−5.77 × 10^−19^ ± 0.046) (*p* = 4.78 × 10^−19^) (Figure 11B). In the case of GPX, infested-untreated plants (0.327 ± 0.105) showed significantly higher expression than the non-infested plants (1.15 × 10^−18^ ± 0.101), while the infested-treated plants (0.273 ± 0.072) did not differ significantly from either treatment (*p* = 3.38 × 10^−13^) (Figure 11C).

At the second sampling date, SOD expression differed significantly among treatments (*p* = 2.89 × 10^−9^). Infested-untreated plants showed the highest expression (0.120 ± 0.014), followed by non-infested plants (5.78 × 10^−19^ ± 0.008), while infested-treated plants showed the lowest value (−0.073 ± 0.025); all pairwise treatment comparisons were significant (Figure 11A). CAT expression was also significantly affected by treatment (*p* = 1.26 × 10^−22^). Infested-untreated plants showed the highest expression (0.220 ± 0.011), non-infested plants remained close to zero (−3.47 × 10^−18^ ± 0.018), and infested-treated plants showed a slightly negative value (−0.050 ± 0.007); all treatments differed significantly from each other (Figure 11B). GPX expression differed significantly among treatments (*p* = 1.41 × 10^−19^): both infested-treated (0.517 ± 0.015) and infested-untreated plants (0.480 ± 0.012) showed higher expression than non-infested plants (−4.63 × 10^−18^ ± 0.027), while no significant difference was detected between the two infested treatments (Figure 11C).

By the final sampling date, SOD expression remained significantly different among treatments (*p* = 1.48 × 10^−12^). Infested-untreated plants showed the highest value (0.157 ± 0.009), followed by infested-treated plants (0.107 ± 0.008), whereas non-infested plants remained near zero (0.00 × 10^0^ ± 0.011); all pairwise comparisons were significant (Figure 11A).

CAT expression also differed significantly (*p* = 1.05 × 10^−17^). Infested-untreated plants showed the highest expression (0.237 ± 0.015), infested-treated plants showed a negative value (−0.087 ± 0.016), and non-infested plants remained close to zero (1.73 × 10^−18^ ± 0.012); all pairwise comparisons were significant (Figure 11B). GPX expression differed significantly among treatments (*p* = 2.16 × 10^−20^). Infested-treated plants showed the highest expression (0.430 ± 0.013), followed by infested-untreated plants (0.293 ± 0.006), while non-infested plants remained near zero (2.31 × 10^−18^ ± 0.019); all pairwise comparisons were significant (Figure 11C).

**Figure 12:**
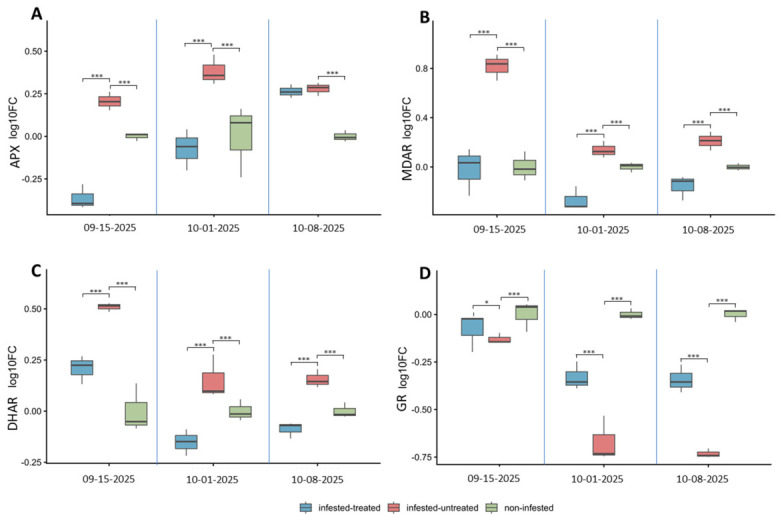
Temporal changes in antioxidant gene expression within treatments in sugar beet leaves in response to insect infestation and treatment. Relative transcript levels of APX (**A**), MDAR (**B**), DHAR (**C**), and GR (**D**) are shown as log_10_ fold-change (log_10_FC) values across three sampling dates (15 September, 1 October, and 8 October 2025). Three plant conditions were compared: infested–treated, infested–untreated, and non-infested plants. Gene expression levels were calculated using the efficiency-corrected 2^−ΔΔCt^ method and normalized to the reference gene actin (ACT). Boxplots represent biological replicates (n = 3), with the median indicated by the horizontal line within each box, boxes representing the interquartile range, and whiskers indicating the full data range. Each biological replicate consisted of pooled leaf samples collected from individual plants per treatment and sampling date. Statistical differences were evaluated within each treatment across sampling dates using robust ANOVA (HC3) followed by estimated marginal means (emmeans) pairwise comparisons. Significant differences are indicated by horizontal brackets with asterisks (* *p* < 0.05, ** *p* < 0.01, *** *p* < 0.001), while non-significant comparisons are not displayed.

At the first sampling date treatment-related differences were observed among the genes of the ascorbate–glutathione cycle. APX expression was significantly higher in infested-untreated plants (0.203 ± 0.056) than in both infested-treated (−0.363 ± 0.074) and non-infested plants (6.62 × 10^−17^ ± 0.027), while the latter two treatments did not differ from each other (*p* = 1.96 × 10^−27^) (Figure 12A). A similar pattern was detected for MDAR, where infested-untreated plants (0.817 ± 0.106) showed significantly higher expression, whereas both infested-treated (−0.020 ± 0.189) and non-infested plants (1.03 × 10^−17^ ± 0.121) remained at comparably low levels (*p* = 1.11 × 10^−25^) (Figure 12B). DHAR followed the same general trend: infested-untreated plants (0.510 ± 0.020) exhibited significantly higher expression than the other treatments, while infested-treated plants (0.207 ± 0.072) showed intermediate values and non-infested plants (3.33 × 10^−3^ ± 0.119) the lowest levels (*p* = 3.08 × 10^−26^) (Figure 12C). In contrast, GR expression also differed significantly among treatments (infested-treated −0.080 ± 0.104; infested-untreated −0.130 ± 0.026; non-infested −3.31 × 10^−17^ ± 0.078) (*p* = 5.61 × 10^−8^) (Figure 12D).

At the second sampling date the overall structure of the responses remained similar. APX expression continued to be significantly higher in infested-untreated plants (0.383 ± 0.086), while infested-treated (−0.073 ± 0.123) and non-infested plants (9.93 × 10^−17^ ± 0.206) showed comparable levels and did not differ statistically (*p* = 7.24 × 10^−17^) (Figure 12A). MDAR displayed a similar pattern, with the highest expression detected in infested-untreated plants (0.137 ± 0.067), the lowest in infested-treated plants (−0.270 ± 0.095), and intermediate values in the non-infested treatment (−8.28 × 10^−17^ ± 0.036) (*p* = 2.64 × 10^−17^) (Figure 12B). For DHAR, infested-untreated plants (0.153 ± 0.110) again showed significantly higher expression than both infested-treated (−0.153 ± 0.065) and non-infested plants (3.33 × 10^−3^ ± 0.050) (*p* = 9.72 × 10^−14^) (Figure 12C). In contrast, GR expression followed an opposite tendency, as infested-untreated plants (−0.670 ± 0.122) exhibited significantly lower expression than the other treatments, while infested-treated (−0.333 ± 0.073) and non-infested plants (−2.81 × 10^−16^ ± 0.026) remained similar (*p* = 1.00 × 10^−28^) (Figure 12D).

By the final sampling date some shifts in the expression patterns became visible. APX expression did not differ between infested-treated (0.263 ± 0.035) and infested-untreated plants (0.280 ± 0.036), but both treatments showed significantly higher expression than the non-infested plants (3.31 × 10^−17^ ± 0.036) (*p* = 1.67 × 10^−27^) (Figure 12A). MDAR maintained the earlier tendency, with infested-untreated plants (0.210 ± 0.080) showing significantly higher expression than both infested-treated (−0.157 ± 0.102) and non-infested plants (9.93 × 10^−17^ ± 0.030), which remained statistically similar (*p* = 7.34 × 10^−16^) (Figure 12B). In the case of DHAR, both infested-treated (−0.087 ± 0.038) and infested-untreated plants (0.153 ± 0.042) showed significantly higher expression than the non-infested plants (−0.003 ± 0.037), while no significant difference was detected between the two infested groups (*p* = 2.24 × 10^−21^) (Figure 12C). GR expression again differed significantly among treatments, with infested-untreated plants (−0.730 ± 0.026) showing lower expression than the other two treatments, whereas infested-treated (−0.343 ± 0.076) and non-infested plants (−1.16 × 10^−16^ ± 0.035) did not differ from each other (*p* = 2.54 × 10^−45^) (Figure 12D).

#### 3.5.2. Temporal Dynamics of Antioxidant Gene Expression

The temporal patterns of gene expression revealed three consistent trends across the experiment. Genes involved in primary ROS detoxification, including SOD, CAT, and APX, already showed significant responses at the first sampling date in infested plants (SOD *p* = 5.68 × 10^−6^; CAT *p* = 0.0018; APX *p* = 2.52 × 10^−9^). As the experiment progressed, these responses remained significant, particularly in the infested-untreated treatment. In parallel, genes associated with the ascorbate–glutathione cycle (DHAR, MDAR, and GR) exhibited strong temporal effects in infested plants. DHAR showed a marked decline over time (*p* = 1.78 × 10^−8^), while MDAR and GR also displayed significant temporal variation (MDAR *p* = 3.41 × 10^−8^; GR *p* = 2.16 × 10^−7^). No significant temporal changes were detected in non-infested plants for any of the examined genes.

### 3.6. Correlation and Principal Component Analysis

**Figure 13:**
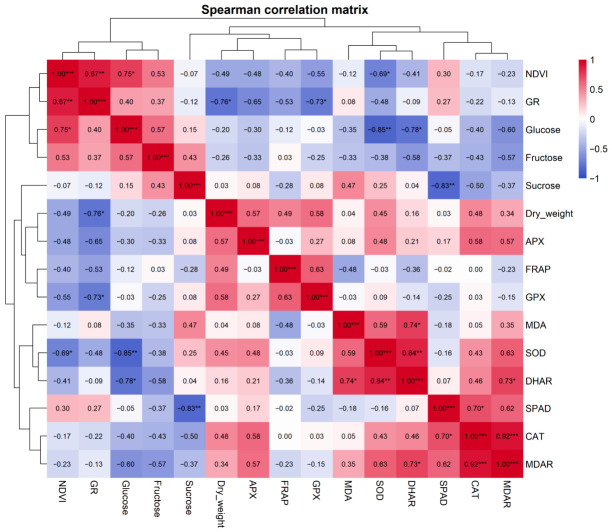
Spearman correlation matrix illustrating the relationships among physiological traits (NDVI, SPAD, dry weight), soluble carbohydrates (glucose, fructose, sucrose), oxidative damage markers (MDA), antioxidant capacity indicators (FRAP), and antioxidant enzyme activities (APX, GPX, SOD, CAT, GR, DHAR, MDAR) measured in sugar beet plants subjected to different herbivory treatments. Correlation coefficients (ρ) are shown within the cells, with colour intensity representing the strength and direction of the relationship (blue: negative correlation; red: positive correlation). Asterisks indicate statistically significant correlations (* *p* < 0.05; ** *p* < 0.01; *** *p* < 0.001). Hierarchical clustering of both rows and columns highlights groups of variables exhibiting similar correlation patterns.

Spearman correlation analysis revealed a structured interaction network linking physiological performance, carbohydrate metabolism, biomass accumulation, and antioxidant defense processes (Figure 13). Hierarchical clustering of the heatmap separated the variables into two main functional groups: parameters associated with plant physiological performance and primary metabolism (NDVI, GR, glucose, and fructose) and variables related to oxidative stress responses and antioxidant defense. NDVI showed the strongest positive correlation with glutathione reductase activity (GR) (ρ = 0.87, *p* < 0.01) and positively correlated with glucose (ρ = 0.75, *p* < 0.05) and moderately with fructose (ρ = 0.53). In contrast, NDVI showed negative correlations with several oxidative stress-related parameters, including APX (ρ = −0.48), FRAP (ρ = −0.40), GPX (ρ = −0.55) and SOD (ρ = −0.69, *p* < 0.05). Furthermore, glucose and fructose showed a moderate positive correlation (ρ = 0.57). Sucrose exhibited weak correlations with most variables but showed a strong negative correlation with SPAD (ρ = −0.83, *p* < 0.01). However, several strong correlations were observed within the antioxidant defense network.

The strongest positive relationship occurred between CAT and MDAR (ρ = 0.92, *p* < 0.001). Strong positive correlations were also detected between SOD and DHAR (ρ = 0.84, *p* < 0.01) and between MDA and DHAR (ρ = 0.74, *p* < 0.05). MDA also showed a positive relationship with SOD (ρ = 0.59). Biomass accumulation (dry weight) showed moderate positive correlations with several antioxidant parameters, including APX (ρ = 0.57), GPX (ρ = 0.58) and FRAP (ρ = 0.49). In contrast, dry weight was negatively correlated with GR (ρ = −0.76, *p* < 0.05) (Figure 13).

**Figure 14:**
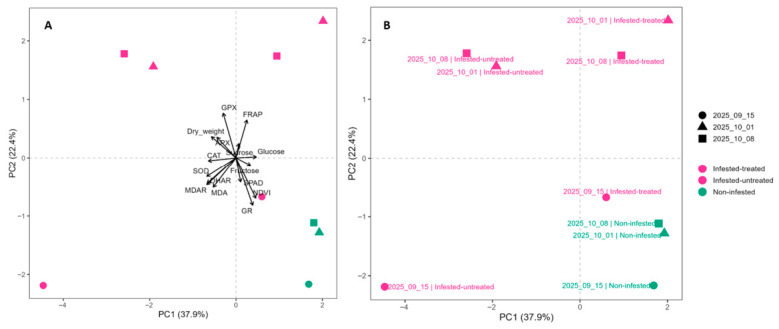
Principal component analysis (PCA) integrating physiological traits, carbohydrate concentrations, oxidative stress indicators, and antioxidant enzyme activities measured in sugar beet plants across three sampling dates (15 September, 1 October, and 8 October). (**A**) PCA biplot showing the orientation and relative contribution of variables to the first two principal components. Vectors indicate the direction and strength of the relationships between variables and the principal component axes. (**B**) PCA score plot illustrating the distribution of individual samples in the multivariate space according to treatment (non-infested, infested-treated, and infested-untreated) and sampling date. The first principal component (PC1) explains 37.9% of the total variance, while the second component (PC2) explains 22.4%. The two axes capture the major patterns of coordinated physiological, metabolic and oxidative stress responses associated with herbivory and treatment effects.

A significant effect of treatment on the multivariate response was revealed by PERMANOVA analysis at early sampling stages. On 15 September, treatment explained 99.7% of the total variance (R^2^ = 0.997, *p* = 0.048), indicating a strong separation of treatment groups. Similarly, on 1 October, treatment remained a dominant factor (R^2^ = 0.999, *p* = 0.032). In contrast, by 8 October, although the proportion of explained variance was still high (R^2^ = 0.900), the effect of treatment was no longer statistically significant (*p* = 0.067). Principal component analysis (PCA) revealed clear treatment-dependent clustering of samples (Figure 14A). Plants that were not infested were always in the positive region of PC1, with scores between +1.5 and +2.0. Plants that were infested but not treated were always in the negative region of PC1, with the lowest score in the dataset (PC1 ≈ −4.5 on 15 September). Samples from the infested-treated treatment occupied an intermediate region of the PCA space, typically ranging between −0.5 and +1.0 along PC1. Temporal dynamics were also evident in the PCA distribution. Samples collected on 15 September exhibited the greatest dispersion along PC1, particularly within the infested-untreated treatment. Samples from October 1 and October 8, on the other hand, formed tighter clusters within each treatment group. This shows that there was less multivariate variability at later sampling dates (Figure 14B). Overall, the PCA score distribution separated treatments primarily along PC1, while temporal effects were reflected in the dispersion pattern of the samples across the PCA space.

## 4. Discussion

The interpretation of the individual phenotypical and oxidative-stress related parameters combined with PCA and correlation analysis indicated that *S. ocellatella* infestation was associated with a metabolic shift affecting photosynthetic performance, carbohydrate metabolism, and redox regulation, with a temporal attenuation of treatment-related differences over time. As a result, the initially strong separation among treatments gradually diminished over time, suggesting a partial stabilization of the physiological and metabolic state of the plants.

The samples were collected during the late vegetative stage of sugar beet, in the period preceding harvest. The temporal structure of the PCA indicates that the strongest metabolic perturbation occurred during the early phase following herbivore attack [40,41]. In plant defense reactions against herbivores, SAR often refers to Systemic Acquired Resistance, which describes the ability of plants to enhance their defensive readiness throughout the entire organism following a local attack [41], The broader dispersion of the 15 September samples may reflect a rapid and heterogeneous metabolic response to herbivory, whereas the more compact clustering observed in later sampling dates indicates partial stabilization of physiological and metabolic processes over time, which in this case could be a sign of increasing resistance against the herbivory of *S. ocellatella* [41], which may be associated with acclimation processes under continued herbivory.

Herbivore damage is known to induce rapid production of superoxide radicals (O_2_^−^), which are subsequently converted to hydrogen peroxide (H_2_O_2_) by superoxide dismutase (SOD). Hydrogen peroxide acts as a relatively stable signalling molecule capable of diffusing across cellular compartments and triggering downstream defence responses. The strong associations observed between SOD, DHAR and MDAR in the correlation matrix, together with their close spatial clustering in the PCA space, indicate that this ROS signalling cascade is tightly coupled to the activation of the ascorbate–glutathione detoxification pathway.

The temporal organization of these responses supports a structured progression of stress effects. First, oxidative imbalance and lipid peroxidation emerge first as the MDA results showed, followed by modulation of antioxidant capacity and antioxidant gene expressions. The positive associations between MDA and antioxidant enzyme genes suggest that increased lipid peroxidation is accompanied by activation of ROS-scavenging mechanisms, reflecting a feedback response of the antioxidant system to oxidative damage that was already proven by excess element supply [17] and also for biotic stressors [42]. The decrease in FRAP and MDA values shows a transition from an early oxidative stress phase toward physiological acclimation. Following the initial reactions induced by herbivory, antioxidant systems appear to mitigate ROS accumulation, leading to a gradual stabilization of redox status. This is supported by the temporal activation of genes of ascorbate–glutathione cycle, indicating a shift from immediate stress response toward longer-term redox regulation and recovery processes. The results of analytical methods (MDA, FRAP, antioxidant gene expression) provide deeper insights into the pest damage through the evident physiological changes that can be traced back to the damage [5,43,44].

The strong positive link between NDVI and GR indicates that maintenance of photosynthetic activity is closely related to the glutathione-dependent redox system, which plays a central role in maintaining cellular redox homeostasis. Furthermore, GR is present in chloroplasts (GR2 isoform), where it protects the photosynthetic apparatus from light-induced damage [45]. Therefore, high GR activity may also aid in maintaining homeostatic NDVI values under herbivore attack [46]. Conversely, the negative correlations between NDVI and various antioxidant parameters suggest that the activation of ROS-detoxifying systems primarily occurs in tissues with diminished physiological performance. This pattern is consistent with stress-induced oxidative signaling, where declining photosynthetic activity is accompanied by enhanced ROS formation and subsequent activation of antioxidant defense pathways. The work of Solano-Alvarez [47] revealed that infection or herbivory leads to lower NDVI values, along with the rise in the activity of antioxidant enzymes such as catalase (CAT), superoxide dismutase (SOD), and glutathione reductase (GR) [48]. A similar pattern was observed in tomato plants (*Solanum lycopersicum* L.) infected with *Clavibacter michiganensis*, where a negative correlation was found between NDVI and the activity of defense enzymes, such as phenylalanine ammonia-lyase (PAL), during the early stage of infection. SPAD values, however, remained stable across treatments and sampling dates, indicating that SPAD is a relatively low-sensitivity parameter of infestation. The transcriptional activation of antioxidant treatments (Figure 8) likely reflects a combination of biological and methodological factors rather than a true lack of physiological impact. Measurements were conducted during the late vegetative stage, when chlorophyll content is relatively stabilized, reducing the sensitivity of SPAD to detect infestation-induced changes. Also, SPAD primarily captures chlorophyll concentration in intact leaf lamina, whereas *S. ocellatella* larvae feed predominantly within internal tissues (petiole and crown region). This internal feeding strategy limits direct damage to the photosynthetically active mesophyll surface. In contrast to SPAD, parameters reflecting functional and metabolic status (NDVI, MDA, FRAP, and gene expression) showed clear treatment responses, indicating that infestation primarily affects physiological performance and redox homeostasis rather than chlorophyll content. Together, these findings suggest that SPAD is a relatively low-sensitivity indicator for this type of herbivory, particularly under late-season conditions and in cases of internal tissue damage.

The CAT–MDAR association indicates a functional link between H_2_O_2_ detoxification and the ascorbate–glutathione cycle. Synchronization between CAT and the ascorbate–glutathione cycle determines recovery or progression to cell death [49].

Similarly, the positive correlations between SOD and DHAR. Superoxide dismutase (SOD) converts superoxide radicals into hydrogen peroxide (H_2_O_2_), which is subsequently detoxified by ascorbate peroxidase (APX), during which ascorbate becomes oxidized to dehydroascorbate (DHA). DHAR then regenerates ascorbate from dehydroascorbate via reduced glutathione (GSH) ensuring sustained ROS detoxification and preventing oxidative collapse [50].

Results indicate sequential activation of oxidative stress responses, first detectable in NDVI and biochemical markers. Lipid peroxidation increased rapidly under *S. ocellatella* infestation, indicating that membrane damage is among the first detectable physiological consequences of larval feeding. This early membrane-level disruption was paralleled by pronounced changes in total antioxidant capacity, both reflecting a rapid adjustment of redox homeostasis [42]. Together, these findings point to an enhanced disturbance of the cellular oxidative balance during infestation. In contrast, leaf dry weight exhibited a delayed response pattern, since significant treatment differences became evident only after prolonged infestation pressure, suggesting that measurable impairment requires the accumulation of oxidative and structural disturbances over time. In this sense, dry weight changes reflect a downstream consequence of sustained stress rather than its initiation.

The gradual nature of the damage induced by *S. ocellatella* in sugar beets was confirmed by the examination of the data collected during the subsequent surveys [51]. If appropriate control measures are not implemented, the damage will persist in the stored beet prism. It was confirmed that the timely application of pyrethroid insecticides containing (contact) insecticides against the pest successfully blocks the progression of the damage. The use of systemic preparations instead of these pesticides poses a food safety risk [7,52].

Plant carbohydrate metabolism is sensitive to both biotic and abiotic stress factors. In sugar beet, infections such as *Cercospora beticola* leaf spot can significantly modify sugar metabolism and photosynthetic activity, resulting in altered levels of sucrose, glucose, and fructose [53]. Sugars also function as osmoregulators and stress-protective metabolites that contribute to cellular water balance and stress adaptation [54]. Accordingly, stress conditions may alter carbohydrate metabolism in sugar beet and modify the ratio of sucrose and reducing sugars.

Leaf-feeding pests can strongly influence carbohydrate metabolism because leaf damage reduces photosynthetic activity and disturbs sugar allocation. The larvae of *S. ocellatella* feed within the leaf petiole, causing considerable damage that may ultimately reduce yield and sugar content [55,56,57]. Fructose and glucose are direct products of sucrose hydrolysis, and their concentrations therefore reflect the regulation of sucrose metabolism [58]. Previous studies have shown that biotic stress modifies invertase activity and consequently alters the concentration of reducing sugars [59,60]. During infections, sugars often accumulate in plant tissues as part of both plant defense responses and pathogen carbon demand [61].

In contrast to the ~2% reduction in sugar content [5,10], no significant differences in total sugar content were detected in the present study. This discrepancy can be explained by several interacting factors. Measurements were conducted during the late vegetative stage, shortly before harvest, when sucrose accumulation in the storage root is largely stabilized. At this stage, biotic stress caused by larval feeding is less likely to induce detectable changes in sucrose content, even if physiological and metabolic alterations are already present at the leaf and cellular levels. While sucrose remained stable, significant changes were observed in glucose and fructose levels, indicating that carbohydrate metabolism was affected. The reason for these findings may lie in the nature of damage caused by *S. ocellatella* larvae that feed primarily within internal tissues (petiole and crown region), which mainly affects carbohydrate transport and source–sink relationships rather than directly reducing the overall sucrose pool in the root. Accordingly, localized and transient changes were observed in reducing sugars (glucose and fructose), while sucrose, as the main storage form, remained relatively stable [62]. Giaquinta [63] were led to this finding regarding the long-term partitioning of sucrose. Experiments using radioisotopes show that sucrose is rapidly transported into the storage root and partitioned into the vacuole with very little conversion to other products [63]. Also, the most severe economic impact of *S. ocellatella* typically occurs during post-harvest storage rather than under field conditions and the substantial loss of sugar content also occurs during storage as well. According to Bazazo and Mashaal [57], the larvae of *S. ocellatella also* damage sugar beets during the post-harvest period. During the research, the pest was detected on harvested roots that had been stored in piles at the edge of the field for several days prior to transport to the processing plant. Tissue damage caused by larval feeding predisposes roots to increased respiration rates, secondary microbial infections, and progressive tissue degradation during storage, all of which can lead to substantial sugar losses. Since our study focused on pre-harvest conditions, these cumulative post-harvest effects were not captured in the measured sugar content. In our experimental setup, the intensity and duration of infestation likely did not reach the threshold required to produce significant changes in total sugar content. Overall, our results suggest that herbivory primarily induces a redistribution of carbohydrate metabolism and modifies source–sink dynamics, while reductions in total sugar content become more pronounced at later stages, particularly during storage.

In the present study, glucose concentrations were significantly lower in infested, untreated plants than in non-infested plants, most likely due to damage that limited the formation of reducing sugars. Similar responses have been reported for pathogen infections where leaf damage induced a reorganization of carbohydrate metabolism [52]. Sucrose accumulation is also frequently observed under biotic stress and is linked to carbohydrate redistribution and stress responses [52,59,60], resulting in a source-sink inversion: leaf damage (e.g., chewing, mining) reduces the photosynthetic surface area. To compensate for this, the plant alters the function of the sugar beet root: it temporarily shifts from a storage organ (sink) to a source. The sucrose stored in the root is mobilized and transported back to the above-ground parts to support new leaf growth and regeneration [52]. In our experiment, sucrose levels increased in infested-treated plants but decreased in infested-untreated plants, suggesting that pesticide treatment supported sugar accumulation.

Herbivory induced strong transcriptional activation of antioxidant genes, indicating rapid redox stress response. At the first sampling date (15 September 2025), several important ROS-scavenging enzymes, such as APX, DHAR, MDAR, and SOD, showed significant treatment effects [57]. In most cases, infested-untreated plants exhibited higher transcript levels than treated plants, indicating that herbivore attack rapidly activated ROS detoxification pathways. This early response in gene activity aligns with the known function of oxidative burst signaling in plant–herbivore interactions, where the buildup of ROS acts as both a direct defense and a signal to activate further defense mechanisms [64]. The results of this investigation are in coordination with the work of ElSayed et al. [65] and further demonstrate that under stress conditions, whether abiotic or biotic, the SOD, CAT, and APX genes show significant upregulation compared with the control.

Additionally, the timing of changes in gene expression indicates that the ascorbate–glutathione cycle is activated in a well-organized manner, which is crucial for maintaining cell balance during stressful situations [66]. Genes involved in this pathway, particularly DHAR, MDAR, and GR, displayed increasingly strong treatment effects over time. According to research, this process is not random but rather a well-organized system in which genes work in synergy to ensure cell survival under stress. Specific genes: DHAR and MDAR are responsible for the regeneration of ascorbate, while GR maintains the reduced state of glutathione, which is essential for DHAR function [50].

Interestingly, not all genes responded with the same temporal pattern, and there is a temporal shift in antioxidant defense mechanisms. While SOD and CAT, which are usually involved in the first response to detoxifying ROS, showed strong reactions early in the experiment, the genes related to glutathione detoxification responded more strongly later on. This change indicates that the process is moving from an initial phase of ROS detoxification to a more complicated stage of redox regulation that includes glutathione-based antioxidant metabolism.

The results of this study should be interpreted within the context of the experimental design. The analyses were conducted across a defined late-season sampling period, providing a targeted snapshot of plant responses under field conditions. While this approach enabled detailed integrative assessment of physiological, metabolic, and molecular parameters, it does not capture the full temporal dynamics across the entire growing season. In addition, transcriptomic data were available for selected sampling dates, which constrained full multivariate integration across all time points. Nevertheless, the consistency of patterns observed across independent datasets and analytical approaches supports the robustness of the identified trends.

It follows that, as a consequence of the future outbreak of the pest in Hungary, the presence of the species will become permanent, with greater damage in these areas. Thus, in order to avoid significant changes in nutrient content, plant protection interventions in sugar beet against this pest, taking into account the criteria of integrated pest management (IPM), will become increasingly important in the future.

## 5. Conclusions

The aim of this study was to assess the progression of *S. ocellatella* infestation in sugar beet near harvest and to evaluate the effect of contact insecticide application. An integrative approach combining non-invasive measurements (CT, SPAD, NDVI), biochemical assays, and gene expression analysis was applied to characterize antioxidant responses. The results indicate that infestation was associated with oxidative stress responses, reflected in increased lipid peroxidation, enhanced antioxidant capacity, and changes in the expression of antioxidant-related genes (SOD, CAT, APX, MDAR, DHAR, GR), particularly in untreated plants. Multivariate analysis suggested associations between physiological performance and antioxidant regulation, with stress responses being more pronounced under reduced photosynthetic activity. While sucrose content remained stable, carbohydrate metabolism was affected, notably by decreased glucose levels. In addition, insecticide treatment was associated with reduced larval damage, as confirmed by CT observations, accompanied by moderated physiological stress responses.

## Data Availability

The raw data supporting the conclusions of this article will be made available by the authors on request.

## Supplementary Materials

The following supporting information can be downloaded at: https://www.mdpi.com/article/10.3390/antiox15050624/s1, Table S1: Results of the normality, homogeneity, and ANOVA tests.

## Abbreviations

The following abbreviations are used in this manuscript:

BBCH	Biologische Bundesanstalt, Bundessortenamt und Chemical Industry
NDVI	Normalized Difference Vegetation Index
CT	Computer Tomography
RT-PCR	Reverse Transcription Polymerase Chain Reaction
FRAP	Ferric Reducing Antioxidant Power
HPLC	High Performance Liquid Chromatography
SPAD	Soil Plant Analysis Development
MAPK	Mitogen-Activated Protein Kinase
MDA	Malondialdehyde
TCA	Trichloroacetic Acid
NADH	Nicotinamide Adenine Dinucleotide (Reduced Form)
